# Use of Workplace Absenteeism Surveillance Data for Outbreak Detection

**DOI:** 10.3201/eid1710.110202

**Published:** 2011-10

**Authors:** Bev Paterson, Richard Caddis, David Durrheim

**Affiliations:** University of Newcastle, Wallsend, New South Wales, Australia (B. Paterson, D. Durrheim);; Transport for London, London, UK (R. Caddis)

**Keywords:** influenza, United Kingdom, surveillance, absenteeism, viruses, Australia, United Kingdom, letter

**To the Editor:** We applaud Mann et al. on their use of a school-based absenteeism surveillance system to compare daily all-causes absenteeism data against a historic baseline to detect outbreaks of influenza-like illness (ILI) as an adjunct to traditional disease reporting (*1*). The growing availability of electronic human resources systems has increased the potential to harness near real-time workplace absenteeism data to complement school absenteeism surveillance and other sources of traditional outbreak surveillance.

In London, United Kingdom, during the first wave of pandemic influenza A (H1N1) 2009, workplace absenteeism data from the Transport for London attendance/absence reporting system were compared with the historical baseline 3-year mean for comparative weeks of the year. The proportion of Transport for London employees absent because of self-reported or medically certified ILI, during June 28–October 17, 2010, generated surveillance alerts when compared with historical baseline data above the 95th and 99th percentile thresholds (SDs 1.96 and 2.58). For the same period, cause-specific workplace influenza absenteeism data were highly correlated with routinely published ILI surveillance, including the National Pandemic Flu Surveillance and sentinel General Practitioner systems (Figure) (*2*).

**Figure:**
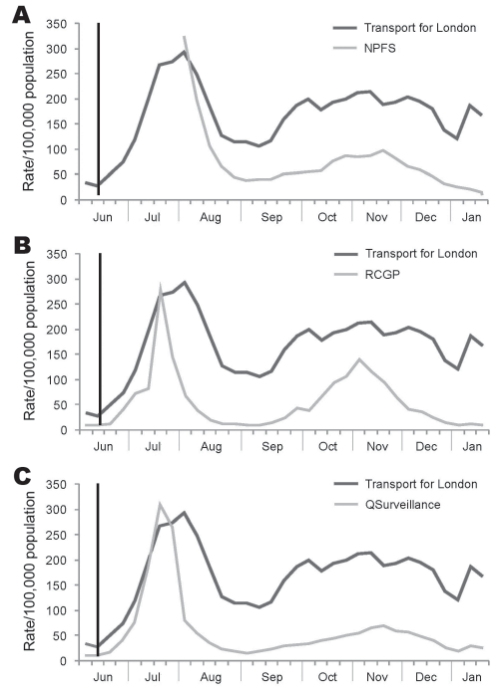
Comparison of transport for London absenteeism rates from influenza data to syndromic surveillance indicators of influenza-like illness rates, London, United Kingdom, 2009. A) National Pandemic Flu Service (NPFS); B) Royal College of General Practitioners (RCGP); and C) QSurveillance. Vertical black line indicates when the World Health Organization declared a pandemic on June 11, 2009. Source: Health Protection Agency, London, and Transport for London.

In Australia, workplace all-causes absenteeism for a major Australia-wide employer has been included as a nonspecific indicator of influenza surveillance by the Australian government for >15 years. A recent study during a severe influenza season in Australia confirmed that employee absenteeism was highly correlated with laboratory-confirmed influenza, and such information could be used to provide surveillance alerts up to 2 weeks before other traditional influenza surveillance data sources (*3*).

The use of workplace absenteeism data, particularly from large employers, has the potential for overcoming the major limitation of school-based absenteeism data in detecting outbreaks of ILI: the effects of school holidays and local planned school closures. Near real-time workplace absenteeism is an effective surveillance tool and should be more widely incorporated in influenza surveillance systems.
